# Endoscopic Excision of Colloid Cysts of the Third Ventricle: Six-Month Outcomes From a Single-Center Series of 22 Patients

**DOI:** 10.7759/cureus.93827

**Published:** 2025-10-04

**Authors:** Talha Sajid, Toqeer Ahmed, Ahmad W Sadiqi, Wajih Ul Hassan, Zia-Ul-Rehman Najeeb, Nasruddin Ansari, Abdul Majid, Asif Bashir

**Affiliations:** 1 Neurological Surgery, Punjab Institute of Neurosciences, Lahore, PAK; 2 Radiology, Lahore General Hospital, Lahore, PAK

**Keywords:** colloid cyst, endoscopic, hydrocephalus, neuroendoscopy, neurosurgery, third ventricle

## Abstract

Objective

The objective of the study is to evaluate the safety, efficacy, and short-term outcomes of endoscopic excision of third-ventricular colloid cysts at a single tertiary care center in a resource-constrained environment.

Methodology

A retrospective review was conducted at the Punjab Institute of Neurosciences, Lahore, from January 2022 to May 2024. The study included 22 patients identified from institutional records including the Picture Archiving and Communication System (PACS) comprising consecutive eligible individuals who underwent endoscopic excision during the study period.

Results

The mean (standard deviation (SD)) age was 34.1 (15.0) years; 12 (54.5%) were female. Headache was the most common presenting symptom, followed by vomiting, drop attacks, and loss of consciousness. Complete excision was achieved in 19 (86.4%) patients. An external ventricular drain (EVD) was placed in 13 (59.1%) patients, and five (22.7%) required ventriculoperitoneal (VP) shunts (two preoperative and three postoperative). Postoperative complications included chemical meningitis in four (18.2%) patients and ventriculitis in two (9.1%) patients; overall, postoperative complications occurred in six (27.3%) patients. One death (4.5%) occurred. At six months, 20 patients completed follow-up; none reported recurrence of primary symptoms.

Conclusion

Endoscopic excision of third-ventricular colloid cysts was feasible in our series, with most patients achieving complete excision and favorable short-term outcomes. Complications and one mortality highlight the need for careful patient selection and standardized perioperative protocols. These results suggest that endoscopy can be a viable option in resource-limited settings, though longer follow-up and larger comparative studies are needed to confirm safety and long-term efficacy.

## Introduction

Colloid cysts are benign, congenital, slowly growing mucoid cysts that comprise 0.5%-2% of primary brain tumors and are typically located at the roof of the third ventricle near the foramen of Monro [1]. These cysts can cause obstructive hydrocephalus by blocking the foramen of Monro and disrupting cerebrospinal fluid (CSF) flow, producing signs of increased intracranial pressure (ICP) such as headache, gait ataxia, nausea, vomiting, and visual disturbance-with headache being the most common [2,3]. Although uncommon, colloid cysts have been reported outside the third ventricle [4].

Because of their deep midline location and proximity to the fornices and deep draining veins, colloid cysts pose neurosurgical challenges [5]. In symptomatic patients, prompt detection on computed tomography (CT) or magnetic resonance imaging (MRI) and timely neurosurgical intervention are essential due to the risk of acute deterioration and, rarely, sudden death [3]. Protein- and cholesterol-rich cysts tend to be hyperdense on CT, hypointense on T2-weighted sequences, and hyperintense on T1-weighted sequences, which has implications for surgical planning [6]. Traditional management includes microsurgical resection via a transcortical or transcallosal approach; neuroendoscopic management has emerged as a viable, safe alternative that may reduce complications in selected patients [7,8].

Colloid cysts of the third ventricle, though rare, pose life-threatening risks due to obstructive hydrocephalus. While endoscopic excision is increasingly preferred, its adoption and outcomes in resource-limited settings are less well characterized. This study aims to evaluate the safety, efficacy, and short-term outcomes of endoscopic excision of third-ventricular colloid cysts at a single tertiary care center in a resource-constrained environment.

## Materials and methods

Study design and setting

We conducted a retrospective review at the Punjab Institute of Neurosciences (PINS), Lahore, Pakistan, covering January 2022 to May 2024. The study included 22 patients identified from institutional records, comprising consecutive eligible individuals who underwent endoscopic excision during the study period.

Inclusion and exclusion criteria

Our study included patients with a third ventricle colloid cyst and ventriculomegaly who presented with symptoms of elevated ICP. All were treated at our center with endoscopic excision, had histopathologic confirmation, and completed at least six months of postoperative follow-up. We excluded individuals with incomplete medical records, those who underwent transcortical excision of colloid cysts, and anyone with less than six months of follow-up.

Data collection

Data were collected from hospital records including the PACS using Google Forms (Google, Mountain View, CA, US). Variables collected included age, sex, residence, comorbidities, presenting symptoms, prior CSF diversion, MRI findings (cyst zone/size, fluid-attenuated inversion recovery (FLAIR) signal), Colloid Cyst Risk Score (CCRS), key operative details (approach, capsule management, hemostasis, and routine septum pellucidotomy), use of an external ventricular drain (EVD), postoperative complications, need for ventriculoperitoneal (VP) shunt, and status at the six-month follow-up. Cyst location was classified according to previously described anatomical zones: zone 1 (anterior third ventricle near the foramen of Monro), zone 2 (mid-body of the third ventricle), and zone 3 (posterior third ventricle) [1].

Complication definitions

Chemical meningitis was defined as postoperative fever accompanied by meningeal signs and CSF pleocytosis in the absence of bacterial growth on culture. Ventriculitis was defined as fever with meningeal signs and CSF pleocytosis with a positive CSF culture consistent with infection.

CSF diversion

Preoperatively, one patient arrived with an existing VP shunt placed at an outside facility, and a second patient underwent emergent VP shunt placement on presentation due to acute obstructive hydrocephalus with neurological deterioration. Intraoperatively and postoperatively, an EVD was inserted when postoperative ventricular bleeding was anticipated or when the operating surgeon deemed it necessary for close monitoring and CSF diversion. Patients who demonstrated persistently elevated intraventricular pressure, sustained high EVD output, or inability to wean from the EVD were subsequently converted to a permanent VP shunt.

Surgical technique

**Figure 1:**
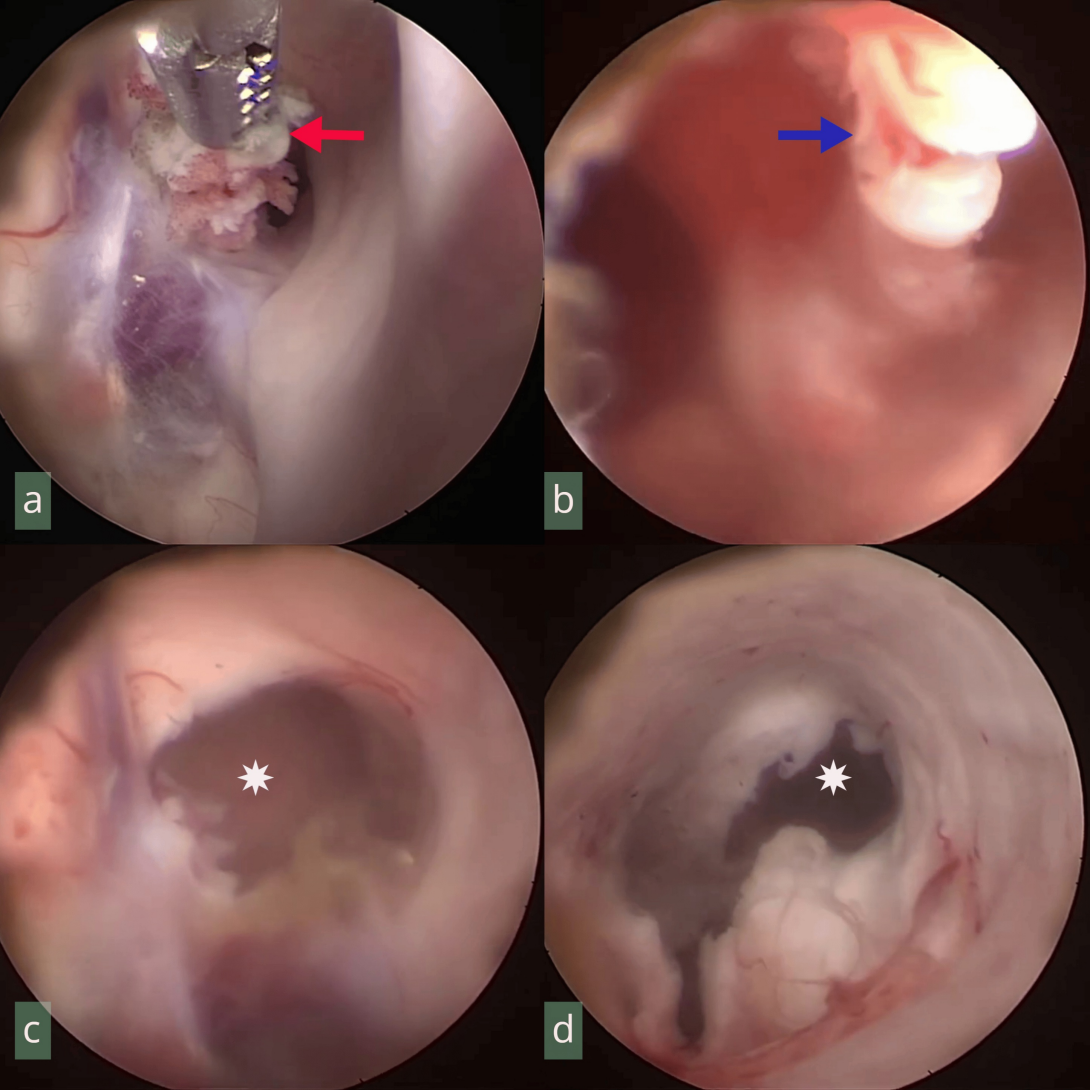
Endoscopic views during excision of a third-ventricular colloid cyst. (a) Forceps grasping the cyst capsule (red arrow). (b) Rotational traction for capsular excision (blue arrow). (c) Foramen of Monro clear of the cyst capsule (white asterisk). (d) Lateral ventricle without active bleeding, roofed by brain parenchyma, during endoscope withdrawal (white asterisk).

A 3 cm linear incision was made, and a burr hole was placed at Kocher’s point (approximately 1 cm anterior to the coronal suture and 2.5-3.0 cm lateral to the midline and 11 cm posterior to the nasion). Under endoscopic visualization, the choroid plexus and/or cyst wall were cauterized as needed, cyst contents were aspirated, and the capsule was gently dissected and excised when feasible using rotational traction and careful dissection (Figure 1). Hemostasis was achieved with continuous irrigation (and bipolar coagulation when required). A septum pellucidotomy was performed in all cases to establish communication between the lateral ventricles, thereby facilitating CSF flow and reducing the risk of unilateral ventricular dilatation or recurrent hydrocephalus following colloid cyst excision, as supported by previous reports [7,9,10].

Data analysis

Analyses were descriptive. Continuous variables (e.g., age, operative time, and length of stay) are summarized as mean ± standard deviation (SD) or median (interquartile range), as appropriate. Categorical variables (e.g., sex, symptoms, prior shunt, and complications) are presented as numbers (percentages). No between-group comparisons or hypothesis testing were performed. Data were summarized using Microsoft Excel (Microsoft Corp., Redmond, WA, US).

Ethics

The Institutional Review Board of the PINS, Lahore, granted an exemption for this retrospective analysis (IRB Exemption #2047/IRB/PINS/Approval/2025). Informed consent was waived due to the use of de-identified records and minimal risk. All images were anonymized, and no patient-identifiable information is included.

## Results

**Figure 2:**
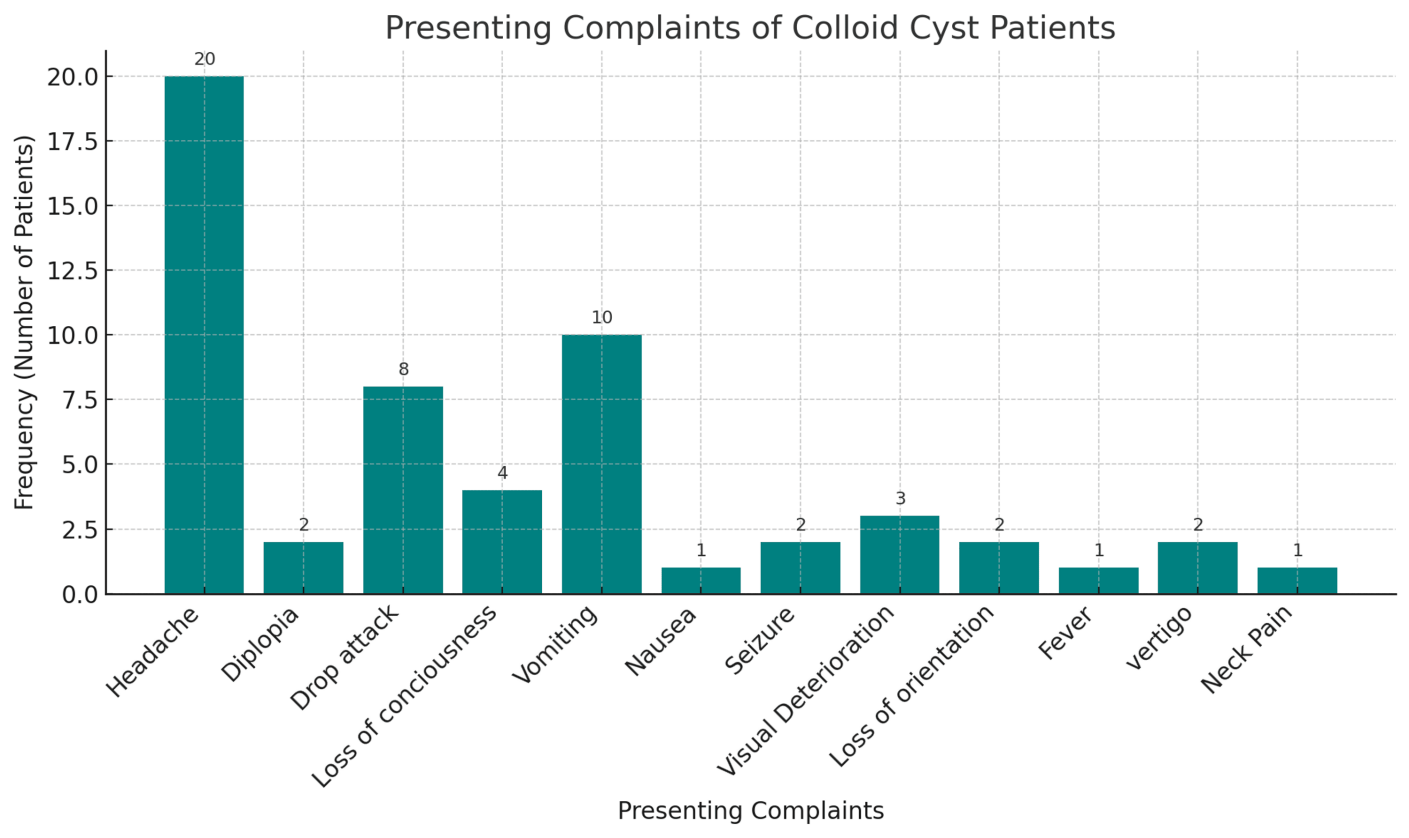
Frequency (n = 22) of presenting complaints of the patients with colloid cysts of the third ventricle. n: total included patients

Of 22 patients, 12 (54.5%) were female and 10 (45.5%) male; mean (SD) age was 34.1 (15.0) years. Rural residence was documented in 17 (77.3%). Hypertension was present in two (9.1%), BMI 20-25 kg/m² in 14 (63.6%), and prior head trauma in one (4.5%). A VP shunt had been placed before cyst surgery in two (9.1%). At admission, Glasgow Coma Scale (GCS) 15 was recorded in 20 (90.9%), GCS 14 in one (4.5%), and GCS 10 in one (4.5%). Headache, present in 20 patients (90.9%), was the most common presenting complaint, followed by vomiting in 10 (45.5%), drop attacks in eight (36.4%), and loss of consciousness in four (18.2%); other symptoms are shown in Figure 2.

**Figure 3:**
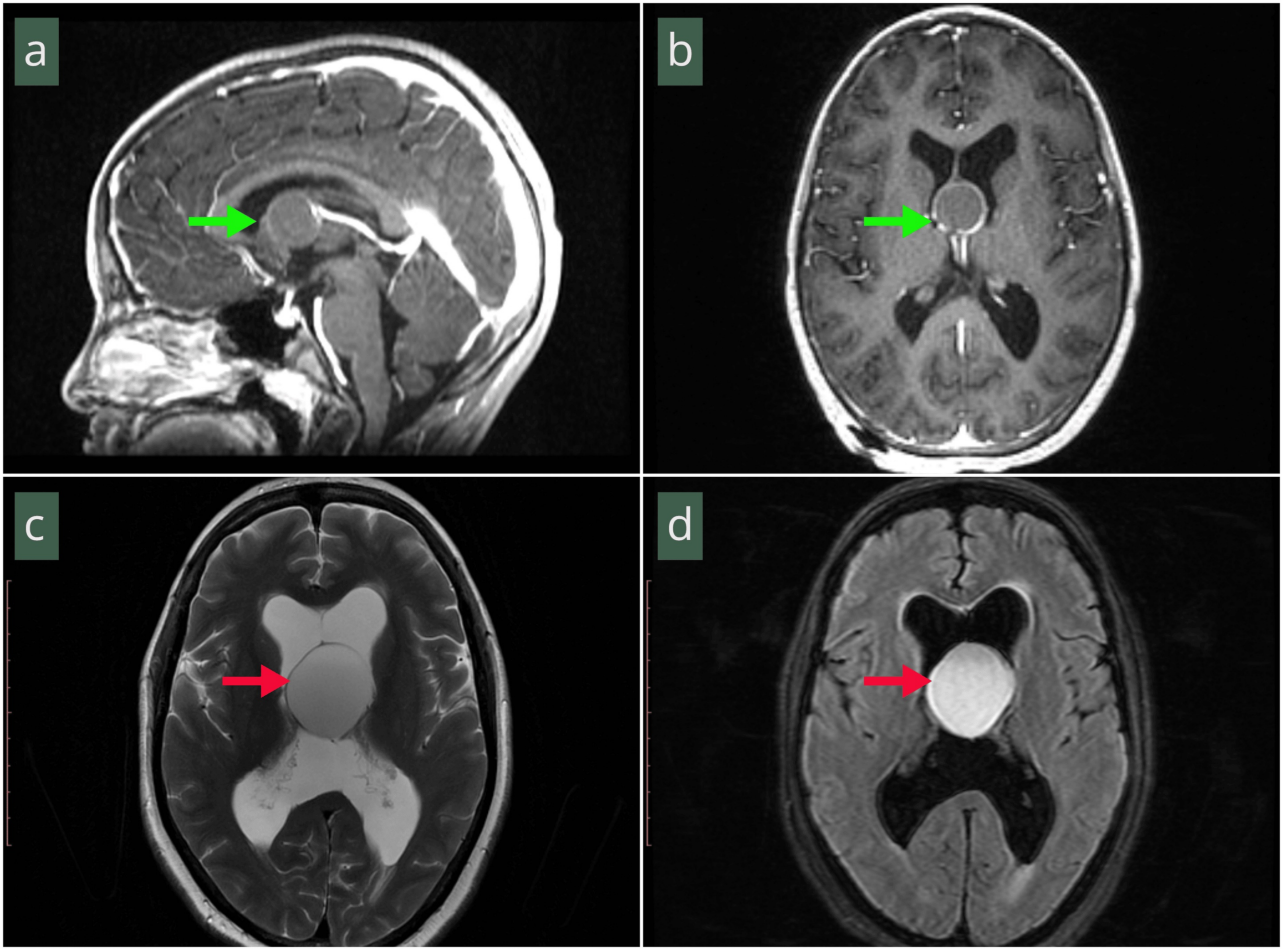
(a, b) Sagittal and axial post-contrast T1-weighted MRI demonstrating thin rim enhancement of the cyst capsule with isointense intra-cystic fluid. (c) Axial T2-weighted MRI showing an iso- to hyperintense, round encapsulated lesion obliterating the third ventricle (red arrow). (d) Axial T2-weighted MRI showing a hyperintense, round lesion obliterating the third ventricle (red arrow). MRI: magnetic resonance imaging

On MRI, T2-FLAIR hyperintensity (Figure 3) was present in seven (31.8%). Cyst location was zone 1 in 14 (63.6%), zone 2 in six (27.3%), and zone 3 in two (9.1%). The CCRS was 5 in seven (31.8%), 4 in five (22.7%), 3 in seven (31.8%), and 2 in three (13.6%).

All patients underwent endoscopic excision under general anesthesia. Complete excision was achieved in 19 (86.4%). An EVD was placed in 13 (59.1%); three (13.6%) subsequently required postoperative VP shunt (from the EVD cohort; 23.1% of those with EVD). Total VP shunt in the cohort was five (22.7%) (two preoperative and three postoperative).

Postoperatively, chemical meningitis occurred in four (18.2%) and ventriculitis in two (9.1%) (with *Acinetobacter* isolated). Overall, postoperative complications occurred in six (27.3%) unique patients. Two (9.1%) required tracheostomy; one (4.5%) patient with ventriculitis and pulmonary complications died. At discharge, 20 (90.9%) had Glasgow Outcome Score (GOS) 5 with GCS 15, and one (4.5%) (tracheostomy) had GOS 3 and was referred for nursing care. Of the two tracheostomy cases, one accounted for the mortality and the other was lost to follow-up. At six months, 20 (90.9%) completed follow-up; no patient reported recurrence of primary symptoms. Memory deficit persisted in two (9.1%). A summary of postoperative outcomes is given in Table 1.

**Table 1:** Postoperative complications and six-month follow-up (n = 22) *Assessed among the 20 patients who completed the six-month follow-up. **This patient was later lost to follow-up. CSF: cerebrospinal fluid; VP shunt: ventriculoperitoneal shunt; GOS: Glasgow Outcome Scale; GCS: Glasgow Coma Scale; EVD: external ventricular drain

Parameter	n (percentage)
Chemical meningitis	4 (18.2%)
Ventriculitis (*Acinetobacter* identified in CSF)	2 (9.1%)
VP shunt placement: total	5 (22.7%)
Preoperative VP shunt	2 (9.1%)
Postoperative VP shunt (from EVD cohort)	3 (13.6%)
Memory deficit (persistent at 6 months)	2 (9.1%)
Mortality	1 (4.5%)
Lost to follow-up	1 (4.5%)
Completed 6-month follow-up	20 (90.9%)
No recurrence/new symptoms at 6 months*	20 (100% of those followed)
Discharge status: GOS 5, GCS 15	20 (90.9%)
Discharge status: GOS 3 (tracheostomy)**	1 (4.5%)

## Discussion

This study presents our experience with endoscopic excision of colloid cysts of the third ventricle, providing insights into patient demographics, clinical presentation, surgical outcomes, and complications. The demographic profile of our cohort revealed a mean age of 34.1 years (SD 15.0), positioning between previously reported ranges in the literature. This age distribution is closer to younger fatal-case series than older elective cohorts [9,11]. Our series demonstrated a slight female predominance (54.5%) and a high proportion of rural patients (77.3%). These findings are most likely reflective of the referral patterns and catchment area of our tertiary care center rather than indicating true epidemiological trends. Given the retrospective single-center design and modest sample size, these demographic observations should be interpreted with caution. The presence of pre-existing conditions such as hypertension in 9.1% of cases and the predominance of normal BMI (63.6% between 20 and 25) provide additional context for patient profiling in colloid cyst cases.

The clinical presentation in our series strongly correlates with previous literature regarding symptom patterns. Headache in 90.9% of our patients closely matches prior reports, with additional symptoms including vomiting (45.5%), drop attacks (36.4%), and loss of consciousness (18.2%) [10,12]. These findings emphasize the potential for acute neurological deterioration, consistent with observations of acute decline in symptomatic cases [10,13]. Sudden death in an eight-year-old girl with a third-ventricular cyst has also been reported, highlighting the potential severity of this pathology [14]. The high proportion of patients presenting with GCS 15 (90.9%) suggests earlier recognition and referral in our cohort, possibly contributing to favorable outcomes.

In our cohort, most cysts were located in zone 1 (63.6%), followed by zone 2 (27.3%) and zone 3 (9.1%). T2-FLAIR hyperintensity was observed in 31.8% of patients. These findings are presented descriptively, and no predictive analysis was performed given the modest sample size. However, previous studies have suggested that cyst zone and radiological characteristics, such as FLAIR hyperintensity, may influence intraoperative complexity, risk of fornix injury, and postoperative outcomes [5,6,15]. Our results, therefore, provide contextual information but should not be interpreted as predictive associations. Risk stratification using the CCRS revealed that 54.5% of our patients scored ≥4 (high risk), 31.8% scored 3 (intermediate risk), and 13.6% scored 2 (low risk). Although CCRS was not used to determine surgical eligibility in our series, documenting it provided valuable context for patient counseling and for comparing our cohort with those reported in the literature [16].

Our endoscopic surgical approach achieved complete excision in 86.4% of cases, comparable to previous studies. The need for intraoperative EVD placement in 59.1%, with 23.1% of those subsequently requiring conversion to VP shunt, underscores the importance of careful CSF dynamics management. The total VP shunt rate of 22.7%, including both pre- and postoperative cases, provides important context for surgical planning and patient counseling. Routine septum pellucidotomy in our series was performed to maintain CSF circulation between the lateral ventricles and stabilize ventricular dynamics after cyst removal. This approach has been recommended in prior literature as a preventive measure against postoperative hydrocephalus and ventricular asymmetry [7,9,10]. While its necessity in all cases remains debated, we believe it offers an additional safeguard in resource-limited settings where shunt dependence carries greater risk. Surgical challenges remain intrinsic to this procedure, including the narrow working corridor at the foramen of Monro, risk of fornix injury leading to memory deficits, careful dissection of the cyst capsule to achieve complete excision, and hemostasis in a deep midline location adjacent to draining veins. These technical constraints highlight the importance of surgical expertise and careful intraoperative decision-making in determining outcomes.

Complications in our series presented notable findings, with chemical meningitis occurring in 18.2% and ventriculitis in 9.1%. Overall, postoperative complications occurred in 27.3% of patients, while persistent memory deficits were observed in 9.1%. Our overall complication rate is somewhat higher than those reported in larger series and meta-analyses, where complication rates typically range from 10% to 25% [5-8,17]. Memory deficits in our cohort (9.1%) fall within the 5%-15% range described in previous reports [7,17]. One mortality (4.5%) was recorded, which is higher than the <2% mortality consistently reported in larger endoscopic series and meta-analyses [8,9,17]. These findings suggest that while our complication profile aligns broadly with existing literature, the higher mortality and shunt dependence in our cohort may reflect the modest sample size and the challenges of infection control and perioperative management in resource-limited settings.

Our findings support early intervention in symptomatic patients, particularly those with high risk scores, along with careful consideration of EVD placement during endoscopic surgery. Vigilant monitoring for postoperative complications, especially chemical meningitis and ventriculitis, is essential. Regular follow-up for potential memory deficits has also proven crucial in our practice. Importantly, this study adds evidence from a resource-limited setting, where infection-control challenges and shunt dependence may differ from high-income centers, making the results particularly relevant for similar contexts.

Limitations

It is a retrospective, single-center series with a modest sample size, which limits generalizability. Patients were selected using non-probability convenience sampling, which introduces the potential for selection bias and reduces external validity. Follow-up was limited to six months for most patients, precluding robust assessment of long-term recurrence, shunt dependence, and neurocognitive outcomes. Complication definitions were based on local clinical practice and may not fully align with standardized international criteria. The descriptive analytic approach did not allow for identification of risk factors or statistical comparison with alternative surgical techniques. In addition, some perioperative decisions, including EVD placement, reflected surgeon judgment and institutional practice, which may differ from other settings. Neurocognitive outcomes were not systematically assessed beyond documentation of memory complaints; no formal neuropsychological testing was performed, and subtle deficits may, therefore, have been missed. These factors collectively constrain the strength of the conclusions, and larger multicenter studies with longer follow-up are needed to better define outcomes and risks.

## Conclusions

In our single-center experience, endoscopic excision of third-ventricular colloid cysts was feasible and provided favorable short-term outcomes. Most patients achieved complete excision, and no patient reported recurrence of primary symptoms at six months. At the same time, complications including chemical meningitis and ventriculitis and one mortality underscore the need for careful perioperative management and transparent counseling. These findings support the role of endoscopy as a viable option in appropriately selected patients, particularly in resource-limited settings, while highlighting the importance of standardized protocols and longer follow-up to define durability and risk more precisely.
